# A scoping review of facilitators and barriers influencing the implementation of surveillance and oral cholera vaccine interventions for cholera control in lower- and middle-income countries

**DOI:** 10.1186/s12889-023-15326-2

**Published:** 2023-03-08

**Authors:** Hanna Trolle, Birger Forsberg, Carina King, Oluwatosin Akande, Stephanie Ayres, Tobias Alfvén, Kelly Elimian

**Affiliations:** 1 Department of Global Public Health, Karolinska Institutet, Stockholm, Sweden; 2 Nigeria Centre for Disease Control, Abuja, Nigeria; 3 Exhale Health Foundation, Abuja, Nigeria

**Keywords:** Cholera, Surveillance, Epidemiology, Cholera Vaccines, Scoping review

## Abstract

**Background:**

Cholera still affects millions of people worldwide, especially in lower- and middle-income countries (LMICs). The Global Task Force on Cholera Control (GTFCC) has identified surveillance and oral cholera vaccines as two critical interventions to actualise the global roadmap goals—reduction of cholera-related deaths by 90% and decreasing the number of cholera endemic countries by half by 2030. Therefore, this study aimed to identify facilitators and barriers to implementing these two cholera interventions in LMIC settings.

**Methods:**

A scoping review using the methods presented by Arksey and O’Malley. The search strategy involved using key search terms (cholera, surveillance, epidemiology and vaccines) in three databases (PubMed, CINAHL and Web of Science) and reviewing the first ten pages of Google searches. The eligibility criteria of being conducted in LMICs, a timeline of 2011–2021 and documents only in English were applied. Thematic analysis was performed, and the findings were presented according to the Preferred Reporting Items for Systematic Reviews and Meta-Analyses Extension.

**Results:**

Thirty-six documents met the predefined inclusion criteria, covering 2011 to 2021. There were two themes identified regarding the implementation of surveillance: timeliness and reporting (1); and resources and laboratory capabilities (2). As for oral cholera vaccines, there were four themes identified: information and awareness (1); community acceptance and trusted community leaders (2); planning and coordination (3); and resources and logistics (4). Additionally, adequate resources, good planning and coordination were identified to be operating at the interface between surveillance and oral cholera vaccines.

**Conclusion:**

Findings suggest that adequate and sustainable resources are crucial for timely and accurate cholera surveillance and that oral cholera vaccine implementation would benefit from increased community awareness and engagement of community leaders.

**Supplementary Information:**

The online version contains supplementary material available at 10.1186/s12889-023-15326-2.

## Background

The risk of adverse clinical outcomes, such as hospitalisation and death, following cholera infection, is higher in vulnerable or fragile settings where access to treatment, standard supplies of potable water and basic sanitation are sparse [1]. Cholera remains a significant public health threat globally and indicates a lack of social development and societal inequities disproportionately affecting people in poverty and exacerbating their vulnerability [2, 3]. The estimated number of cholera cases is 2.9 million worldwide and 95,000 deaths per year [4], with 47 lower- and middle-income countries (LMICs), particularly in sub-Saharan Africa and South Asia, contributing most cases [5]. Asia has a long history of endemic cholera, while the disease has increasingly manifested itself in Africa in recent decades [6]. For example, 32 out of 37 states in Nigeria have recorded 103,589 suspected cholera cases between epidemiological week 1 and 46 in 2021, with 3,566 deaths and a case fatality ratio of 3.4% [7].

The relative ease with which the 2021 outbreak spread across Nigeria underlines the importance of meeting the World Health Organization (WHO) Global Task Force on Cholera Control (GTFCC) goals for cholera control. These goals include reducing cholera-related deaths by 90% and decreasing the number of cholera endemic countries by half by 2030. Moreover, cholera control is a core component of achieving the Sustainable Development Goals (SDGs), particularly Goal 3 - “ensuring healthy lives and wellbeing for all” – and Goal 6 - “access to clean water and sanitation for all” [5]. In order to strengthen the response to cholera and achieve the aforementioned goals, the GTFCC has proposed six primary interventions. These interventions include oral cholera vaccine (OCV) use; surveillance (epidemiology and laboratory); healthcare system strengthening; leadership and coordination; community engagement; and improving access to water, sanitation and hygiene. These six primary interventions are the same as the five pillars in the GTFCC’s document on ending cholera by 2030, with the addition of leadership and coordination as the sixth intervention [5]. In this study we focus on the surveillance and OCV pillars.

Surveillance relates to early detection of cholera to guide timely outbreak response and routine collection of epidemiological data to assess disease burden and identify endemic areas and cholera hotspots [5]. Timely and reliable surveillance data on cholera is paramount to detecting outbreaks at an early stage and to monitoring changes and trends in mortality and morbidity. For a surveillance system to function it is crucial to have adequate laboratory capacity for testing and confirming suspected cholera cases [8]. Surveillance informs planning of other interventions, such as OCV [9]. OCV provides a safe, practical and feasible way of protecting populations from cholera [10, 11]. Currently three OCVs (Dukoral, Shanchol and Euvichol) have been pre-qualified by the WHO and all require two doses in order to be fully protective. While Dukoral is commonly used for travel vaccination, the global OCV stockpile used for mass vaccination campaigns during cholera outbreaks and emergencies consists of Shanchol and Euvichol [12]. If OCV is implemented within a community, surveillance is important for measuring the impact of OCV by monitoring trends in cholera cases.

The relationship between surveillance and OCV is important during outbreak management and informed our decision to explore these two interventions concurrently. Evidence on the facilitators and barriers to the implementation of both interventions would serve as a valuable and relevant tool for global, regional and local policymakers, as well as for the GTFCC in planning and implementation of said interventions. Our study aimed to identify the factors influencing the implementation of surveillance and OCV interventions for cholera control in LMICs.

## Methods

We conducted a scoping review, as per the guide presented by Arksey and O’Malley [13]. The five stages of a scoping review in relation to our research question are outlined below:

### Identifying the research question

The specific research questions for the present study were: (1) What are the facilitators that influence the implementation of surveillance and OCVs for cholera control in LMICs? (2) What are the barriers hindering the implementation of surveillance and OCVs for cholera control in LMICs?

### Identifying relevant studies

Identification of relevant documents for this scoping review relied on searching through three research databases (PubMed, Cumulative Index to Nursing and Allied Health Literature (CINAHL) and Web of Science) and the Google website. A search strategy was developed through consultations with a Karolinska Institute librarian. Medical Subject Headings (MeSH) terms were used where possible and were otherwise modified to fit databases that do not use MeSH terms. Several combinations of the search terms were created and the key search terms were *cholera*, *surveillance*, *epidemiology* and *vaccines* (see a summary of the search outputs in Additional file 1). A systematic Google website search was also conducted, using the above mentioned key search terms and screening the first ten pages to find potential documents that may not have been indexed in the previous databases for inclusion. However, no data from the Google search was eventually included for analysis due to duplications. Database searches were conducted on 12 February 2021 and Google search was conducted on 2 and 5 of April 2021. Additional file 2 and 3 present the database and Google search respectively, along with their search terms and outputs.

### Study selection

We used the following inclusion criteria: the study was conducted in an LMIC, as defined per the latest World Bank classification system [14]. Timeline was originally set to anytime in 1990 to February 2021. During the later stages of the screening process the timeline was changed to 2011 to February 2021. This was done on account of there being still too large an amount of data given the limited time for the scoping review and that the GTFCC activities were revitalised during that period [15]. The following inclusion and exclusion criteria were applied in the selection of documents: documents focused on cholera surveillance and/or OCV; LMICs; written in English language; quantitative and/or qualitative in nature; and peer-reviewed (See Table 1 for the list of inclusion and exclusion criteria). The data searches were imported into Rayyan software to aid in sorting and removal of duplicates [16]. After the de-duplication process, title screening was conducted to include documents with titles relevant to the research questions, followed by abstract screening. The last step consisted of full-text screening, after which the final documents were included for analysis.

**Table 1:** Inclusion and exclusion criteria

**Inclusion criteria**
LMICs (5)
English language results
Cholera surveillance
Cholera outbreak
OCV intervention/plan/programme/campaign
OCV acceptance/uptake
Cholera control/response
Cholera in conflicts/emergencies/natural disasters
Quantitative studies
Qualitative studies (interviews, focus groups etc.)
Randomised control trials (RCT)
Cohort studies
Case-control studies
Cross-sectional studies
Peer-reviewed articles
**Exclusion criteria**
High-income countries
Development/production of OCV
Levels of OCV protection (biological and/or immunological factors)
Studies on epidemiology of the cholera bacteria (specific serogroups and biotypes)
Studies on epidemic diseases in general (not specific enough focus on cholera)
Focus on serogroups other than O1 or O139
Studies on cost, cost-effectiveness, Willingness to pay (WTP)
Models, forecasting
Comparing interventions
Cholera in travellers
OCV and pregnancy
Animal, in-vitro studies
Environmental studies
Case studies, case reports
Historical articles
Letters, comments, perspectives, editorials, reports, meeting notes
Systematic and scoping reviews, meta-analyses

### Charting the data

The charting process consisted of structuring the collected data into a Microsoft Excel database, which acted as an extraction form where relevant variables from the selected documents addressing the research questions were charted. Descriptive and methodological indicators were used to categorise the data. There were separate columns for surveillance facilitators and barriers and columns for OCV facilitators and barriers. At this stage, we identified the facilitators and barriers from the included documents in their entirety.

### Collating, summarising and reporting the results

At this stage, the included data were collated, compared and summarised. Then thematic analysis was conducted using the methodology recommended by Braun and Clarke [17]. An inductive approach to data analysis was used, meaning analysis was performed with as few preconceptions as possible of what themes may be identified in the data [17]. In addition to thematic analysis, descriptive indicators were imported to STATA Version 16 Software [18] to describe the selected documents. For the results to be used practically for policy, research or practice, the implications of the findings were discussed.

## Results

### Description of documents

**Fig. 1:**
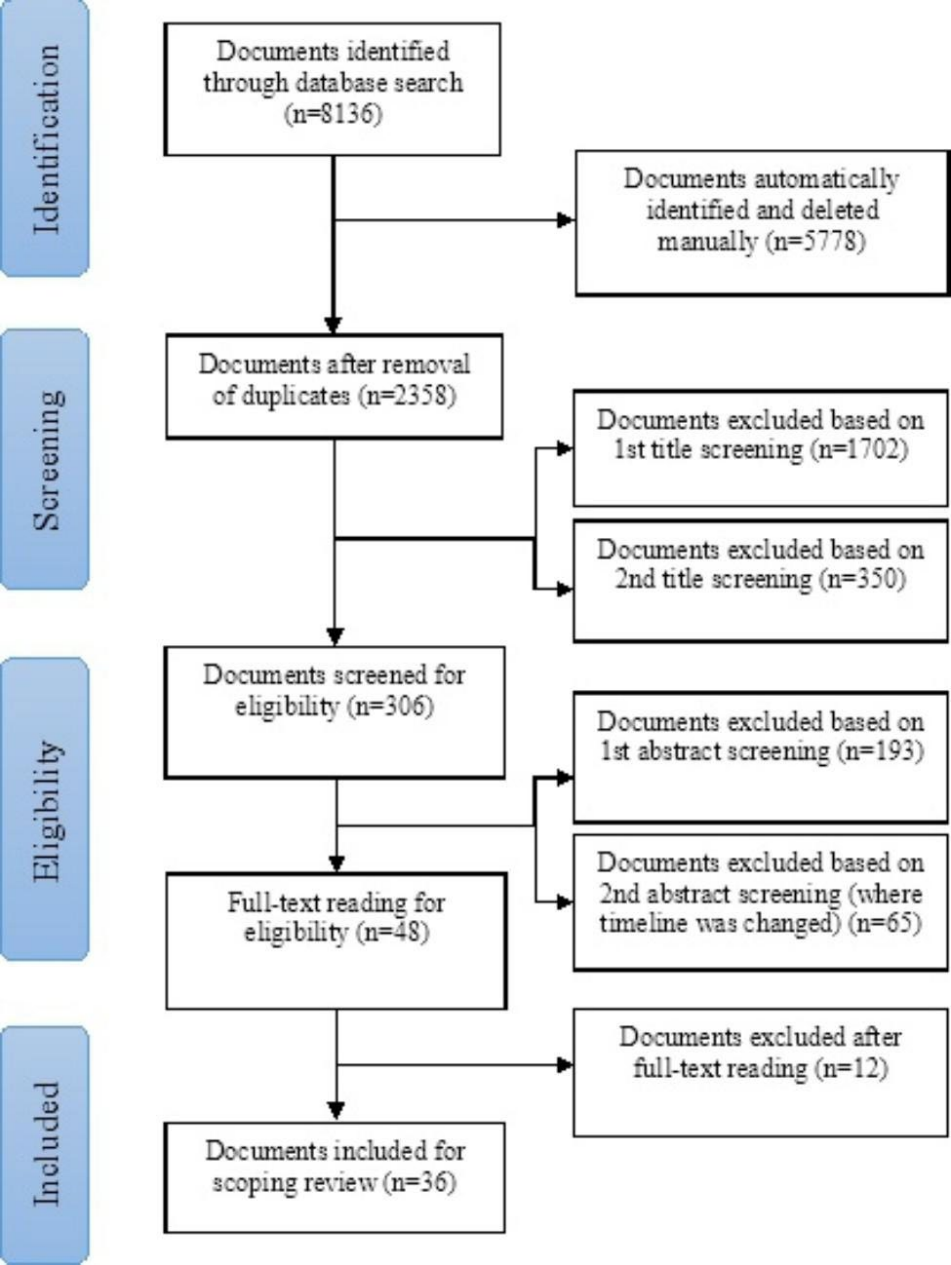
Flowchart of the selection of documents for the scoping review

Figure 1 presents the Preferred Reporting Items for Systematic Reviews and Meta-Analyses – Extension for Scoping Reviews (PRISMA-ScR) flow chart showing the selection process of the analysed documents. A total of 8136 documents were originally identified through searching the three databases, of which 48 documents were read in full and 36 met the predefined inclusion criteria for the study. The characteristics of these are summarised in Table 2. Of the 36 documents, all were from peer-reviewed journals, more than half (67%) were published between 2016 and 2021. As mentioned previously, the timeline of 2011–2021 was used in order to find documents which were published within that timeline. While the documents were all published within said timeline, the documents’ study years span between 2005 and 2019. Most of the documents (56%) focused on cholera in an epidemic context. Haiti was the most studied country with four (11%) studies among the selected documents. Table 3 provides an overview of the identified themes. See additional file 4 for a summary of each of the 36 documents as well as the facilitators and barriers of each document.

**Table 2:** Description of documents analysed for the study

Description of documents analysed for the study		
Descriptive characteristic	Frequency (N = 36)	Percentage (%)
**Year of publication**		
2021 2020 2019 2018 2017 2016 2015 2014 2013 2012	1 5 3 4 6 5 2 4 5 1	3 14 8 11 17 14 6 11 14 3
**First author affiliation**		
Government Academic/Research NGO	16 13 7	44 36 19
**Study design**		
Descriptive Evaluation Intervention Interview (qualitative) Mixed-methods	6 18 3 1 8	17 50 8 3 22
**Serogroup**		
O1 O1, O139 Not stated	6 6 24	17 17 67
**Serotype**		
Ogawa Inaba Ogawa & Inaba Ogawa & El Tor Ogawa, Inaba & El Tor Not stated	3 1 1 1 1 29	8 3 3 3 3 81
**Country**		
Bangladesh Cameroon Democratic Republic of Congo Ghana Guinea Haiti India Iraq Malawi Mozambique Nigeria Somalia South Sudan Thailand Uganda Zambia Zanzibar (Tanzania)	3 2 2 3 1 4 2 1 3 3 3 1 2 1 2 2 1	8 6 6 8 3 11 6 3 8 8 8 3 6 3 6 6 3
**Context**		
Epidemic Routine	20 16	56 44
**Study year**		
2019 2018 2017 2016 2015 2014 2013 2012 2011 2010 2009 2012–2013 2011–2015 2011–2012 2010–2011 2007–2011 2005–2013	1 3 3 6 2 5 2 3 2 2 1 1 1 1 1 1 1	3 8 8 17 6 14 6 8 6 6 3 3 3 3 3 3 3

**Year of publication** - year when included journal articles were published. **Study year** - year when studies in included journal articles were conducted. **First author affiliation** – the type of organisation the first author was affiliated with. **Study design** – the chosen study design of the included journal articles. **Serogroup** – cholera serogroup(s) stated in the included journal articles. **Serotype** – cholera serotype(s) stated in the included journal articles. **Country** – Country where studies of the included journal articles were conducted. **Context** – Studies conducted in the context of a cholera epidemic or outbreak (Epidemic) or studies conducted in the context of a non-epidemic, non-oubreak, routine setting (Routine)

### Thematic analysis

**Table 3:** Overview of identified themes

Cholera intervention	Theme
Surveillance	Timeliness and reporting
Surveillance	Resources and laboratory capability
OCV	Information and awareness
OCV	Community acceptance and trusted community leaders
OCV	Planning and coordination
OCV	Resources and logistics

### Surveillance themes

#### Timeliness and reporting

Timeliness and reporting are crucial to cholera surveillance and response. Timeliness is characterised by early detection, reporting and confirmation of cholera cases, and declaring and responding to cholera outbreaks [19, 20]. Studies in Uganda and Nigeria showed that a surveillance system that is prepared and well-coordinated by prioritising actions and collaborations among stakeholders can facilitate timeliness, with a resultant impact on early response to cholera outbreaks [21, 22]. In contrast, delayed reporting of cholera cases and declaration of cholera outbreaks through a delay in data transmission from lower (e.g. primary) to a higher (specialist or tertiary) levels of healthcare, can have a deleterious impact on the timeliness of cholera surveillance. This scenario was evident in Borno State, Nigeria and Brong Ahafo Region, Ghana [22, 23].

Weak community-based surveillance contributed to the late reporting of cholera cases in Ghana [19]. Additionally, prompt cholera surveillance can be hindered by poor record-keeping, incomplete reporting, incomplete analysis of surveillance data, as well as discrepancies, inaccuracies and missing information on reported cholera cases [19, 21, 24]. Other barriers to prompt cholera surveillance included: inaccurate locations of cholera cases, limiting surveillance efforts to only certain zones and difficulty reaching male cholera patients as they were often away during surveillance activities [23–25]. Furthermore, poor knowledge of cholera surveillance systems on a local level, combined with weak local-level collaboration, such as weak communication between neighbouring communities, created further barriers [19, 21, 24, 26].

#### Resources and laboratory capability

A variety of resources, including human resources (e.g. well-trained staff) [26, 27], financial resources [21], and technical resources (e.g. vehicles and phones), were identified as facilitators of cholera surveillance [22, 26]. Cholera surveillance and laboratory testing capacity is co-dependent on each other, thus leading to aspects of surveillance being connected to resources and laboratory capability. Strong laboratory capabilities, such as expertise in Polymerase Chain Reaction (PCR) testing, availability and readiness of reference laboratories and use of unambiguous cholera case definitions, were also identified as being crucial in facilitating proper cholera surveillance in Mozambique, Cameroon and Ghana [25–27].

However, limited resources as identified above, the absence of an electronic system for reporting, as well as inadequate staff training acted as barriers to cholera surveillance [21, 22, 24–26]. Notably, lack of laboratory capability for confirmation of cholera cases was identified as a recurring barrier to cholera surveillance [22, 25, 26], especially at the district (state) level in Uganda between 2007 and 2011 [21]. Furthermore, the inability of several districts to send adequate numbers of samples to the laboratory for culture confirmation was noted in Ghana in 2014 [23].

### Oral cholera vaccine themes

#### Information and awareness

A recurring facilitator of implementing OCV interventions was pre-existing knowledge of cholera as a disease in the community. Community members’ awareness of the seriousness and symptoms of cholera contributed to their motivation to receive OCV during OCV campaigns [28–32]. Seven of the studies noted that community members’ knowledge and awareness of OCV is an important preventive and protective measure against cholera [31, 33–38]. Studies also noted that having knowledge, awareness and information about actual OCV campaigns being conducted acted as a facilitator of OCV implementation [32, 39–43]. This was evident in Dhaka, Bangladesh, where some persons reported knowing that OCV delivery was taking place at non-governmental organisations (NGOs) working in the area [34]. Providing clear communication on the key messages of an OCV campaign was crucial in Nampula, Mozambique [29], as was correct information around self-administration of a second dose in Lake Chilwa, Malawi, where participants needed to be reminded of when to take the dose [33, 44].

Lack of knowledge of OCV however acted as a barrier to its implementation, an issue that was often characterised by misconceptions, vaccine hesitancy and refusal [29, 30, 33, 41]. In Kalemie, the Democratic Republic of Congo, some community members felt OCV was unsafe, had no effect or that it could cause illnesses [40]. Members of other communities did not trust that the vaccine was authentic [31, 33], feared side effects [45] or questioned the dose recommendations [31]. People in Lusaka, Zambia lacked information on potential OCV side effects, target population and duration of protection. Consequently, observing side effects among community members became a credible rationale for spreading rumours of OCV being unsafe [46]. In Nampula, Mozambique, there was also hesitancy towards oral administration of cholera vaccine in comparison to injections, where the former mode of vaccine delivery approach was seen as perhaps less efficacious as compared to the latter [29]. In some instances, lacking information on OCV led to the perception that vaccines are only meant for children [22, 40, 47].

In a study of attitudes toward OCV in Dhaka, Bangladesh, it was reported that only 16% of 2,830 participating families had heard of OCV [34]. Lack of awareness, not hearing about vaccine activities or date, time and site of the OCV campaign were examples of other barriers to the implementation of OCV - lacking information about the vaccination campaign itself [28, 39]. Innovative strategies of self-administration of OCV were also poorly communicated to the community [33, 44]. A study in Zanzibar noted that if people were away or busy, they were less likely to get information on OCV campaigns on time and were likely to miss out on OCV altogether [45].

#### Community acceptance and trusted community leaders

In several study contexts, a high level of acceptance of OCV was mediated by pre-existing positive attitudes towards and willingness to receive the vaccine by community members [33–36, 48–50]. In Nampula, Mozambique, there were also positive attitudes toward vaccines in general [29], with some of these attitudes having a positive impact on other vaccine campaigns, as in the case of Dhaka, Bangladesh [37, 51]. In other studies, there were instances where community members expressed willingness to get vaccinated again in the future, should there be another OCV campaign [31, 32, 40]. In some cases, in South Sudan and Bangladesh, persons receiving OCV would volunteer to promote vaccination uptake within their communities and recommend others to get vaccinated [31, 37].

Another facilitator of OCV campaigns was having well-trained community leaders [33] and having informed and engaged community stakeholders [30]. Further aspects aiding OCV interventions were community members’ increased trust in vaccine providers [31] and the use of accepted community volunteers and leaders who could conduct OCV campaigning amidst insecurity challenges [42]. In one study in Guinea, vaccine recipients thought it was reassuring that the Ministry of Health and other actors participated in the OCV campaign [32]. In Bangladesh, OCV was perceived as safe by the community since vaccination was implemented by the government [37]. Having a community leader assuring community members of the vaccine’s safety also worked as a facilitator [28]; for example having the commissioner vaccinating in a campaign in Nigeria [22].

The most common OCV information sources were messages through megaphones, local criers, healthcare workers, family and friends, community sensitisation in school, church, home visits, social networks, word of mouth from neighbours, trusted healthcare organisations and announcements inside Internally displaced persons (IDP) camps [35, 42, 43, 46, 49, 52, 53].

However, a reported unpleasant taste and/or smell of the OCV was an often observed barrier [28, 30, 31, 37, 40, 46, 51, 52], which at times led to participants spitting out the vaccine [43], and possibly putting them at risk of receiving incomplete doses of OCV [32]. In some cases, rumours were acting as barriers to OCV interventions. For example, community-level rhetoric in Mozambique furthered the idea that political opponents or enemies used the OCV campaign to hurt the community [29]. Some participants in Bangladesh became reluctant because of the rumour that the people were being used as guinea pigs to test OCV [37].

#### Planning and coordination

A core facilitator for OCV campaigns was adaptive planning and coordination of the actual campaign. Adapting to local contexts included conducting vaccination on weekends, mobile vaccination teams, door-to-door vaccination, using fixed sites, starting vaccination early and finishing late in the evening [32, 41, 46, 49, 53] – all to reach as many persons as possible in as wide an area as possible. OCV campaigns also benefitted from fast response and coordination when cholera needed to be prevented quickly. Moving quickly from the decision to use OCV and requesting delivery from stockpiles to the implementation of a campaign was an important facilitator in countries like Zambia, Nigeria, Iraq and Malawi [20, 22, 41, 50]. Being well-coordinated and having good cooperation among OCV campaign actors were other facilitators identified in Nigeria and Bangladesh [22, 51]. Additionally, by approving OCV use in Nigeria where cholera is endemic, the country was prepared for emergency OCV distribution when an outbreak occurred [22]. Micro-planning facilitated OCV campaigns, as well as planning them in a feasible way [47] that the public responded to and accepted [33]. Micro-planning guides were also adapted from a similar context of Sierra Leone to Borno State in Nigeria [22].

One of the most prominent barriers in several studies was locating eligible recipients of the vaccines during OCV campaigns, as they were often absent from their home, busy, working, travelling or having other commitments [31, 32, 35–37, 39, 41–44, 50, 52, 53]. Thus, at times it was hard to reach working adults, mostly men, with vaccination [40, 51]. A core issue in Nampula, Mozambique, was insufficient planning [28] and in Uganda planning was made difficult because of unclear dates of vaccine shipping [36]. Another aspect making it harder to reach more persons eligible for OCV was vaccination teams missing or not visiting households for various reasons [36, 41, 42].

#### Resources and logistics

A variety of resources facilitated successful implementation of OCV campaigns: use of affordable and easy-to-use vaccines [52]; the existence of vaccine stockpiles [41]; use of vaccination cards during vaccine distribution (each vaccine recipient receives a personal card certifying they have been vaccinated) [28]; and mHealth solutions for vaccine registers to minimise printing and manual paperwork [54]. Having well-trained, experienced and committed human resources also facilitated OCV campaigns [22, 36, 41, 47].

Another facilitator often observed was functioning logistics for proper OCV distribution, such as logistical vaccine management and a functioning cold chain system [28, 47, 48, 55]. In some contexts, this was further facilitated by using pre-existing logistical and cold chain structures previously put in place by polio vaccine campaigns and/or Expanded Programmes on Immunization (EPIs) [22, 42, 47, 50]. Further ways of overcoming logistical hurdles, like vaccine storage, were the delivery of OCV in batches and using a phased release and storage approach during vaccination campaigns [22, 49]. The thermostability of certain OCV, such as Shanchol, was also a facilitator to vaccine administration outside of a cold chain [20, 44, 49]. Where self-administration was feasible, it lowered the logistical burden of a two-dose campaign. Additionally, this was further facilitated by participants’ ability to correctly keep vaccines refrigerated at home [44, 51].

A recurring barrier to OCV was the lack of OCV doses and stockpile shortages [20, 26, 41, 44, 48]. One study in Cameroon described how vaccine shortage created the issue of choosing between conducting a timely OCV campaign with one dose or waiting for available doses to provide a two-dose campaign [48].

Poor implementation of vaccination cards appeared with challenges in South Sudan [49] and in Borno State, Nigeria, where vaccination cards were not being distributed during the first round of vaccination. This because the assigned vaccinators assumed there would not be a second round of vaccination and therefore saw no need for vaccination cards. This was because the assigned vaccinators were used to vaccinate against polio, which only requires one dose [22].

Further resource barriers were complicated OCV vial packaging [22, 47, 49], the flow of vaccination data not working sufficiently due to poor communication networks and inadequate budgeting and financing of OCV campaigns [22].

The main logistical barriers to OCV implementation are unmet cold chain requirements. For example, in humanitarian settings, the need for large storage of single-dose OCV while maintaining the cold chain was difficult to fulfil. Cold chain ruptures further hindered vaccine administration, resulting in loss of vials due to freezing [22, 47, 49, 52]. For some participants in Lake Chilwa, Malawi, and Dhaka, Bangladesh, self-administration of OCV was seen as complicated, with some persons finding it difficult to take the vaccine themselves and worrying about how to store it correctly at home, further demonstrating the logistical barriers to OCV implementation [33, 44, 51].

### The interface between surveillance and oral cholera vaccines

Having proper cholera surveillance is a facilitator to evaluate OCV campaigns [48]. Using surveillance can also allow for the timing of OCV campaigns to seasons when cholera is less common (off-season) [52]. Furthermore, conducting daily and/or nightly reviews of vaccination data and subsequent case-finding facilitates planning and adapting OCV campaigns to local conditions using the collected data. This also promotes further follow-up [54]. In addition, lack of an accurate map of a planned vaccination area, can hinder the possibility of an exhaustive door-to-door OCV campaign [28]. In areas with a dynamic population and lacking population records, it can be hard to accurately estimate how many people have had or will need OCV [20, 35].

## Discussion

This scoping review of 36 documents has identified several facilitators and barriers influencing the implementation of surveillance and OCV for cholera control in LMICs. The themes identified under surveillance were timeliness and reporting, and resources and laboratory capabilities. For OCV, information and awareness, community acceptance and trusted community leaders, planning and coordination, and resources and logistics were identified.

### Interpretation of key findings

A key finding of this review is the importance of accurate and timely information on cholera and OCV delivery from a trusted information source to the community. If potential vaccine recipients do not receive adequate information, it could lead to misinformation and distrust of vaccines, thus hindering the implementation of OCV interventions. A study on possible OCV implementation in Haiti [56] discussed the importance of informing the community about cholera given the population had no previous experience of the disease. The authors’ recommendation was to develop a clear communications strategy ahead of delivery of OCV [56]. This supports the notion that information about OCV campaigns needs to be tailored to local contexts and delivered in a consistent way, in order to reach as many people as possible. The results of the scoping review also demonstrate the importance of capitalising on community members pre-existing knowledge of cholera and OCV. This may provide openings for an information campaign to anchor into a community already harboring positive attitudes toward vaccination against cholera. If people’s queries and worries about OCV function, safety and side effects are not considered when planning an OCV campaign, this may harm OCV uptake among the community with the risk of growing vaccine hesitancy. Furthermore, people want to be reassured that OCV are safe. Involving already accepted community stakeholders and leaders can therefore help an OCV campaign gain trust. This is expressed in another study on an OCV campaign in Haiti. The directors of the vaccinating organisation in Haiti were present throughout the OCV campaign to reassure community members of vaccine efficacy and inform of potential side effects. The work of the organisation benefitted from the trust it had built in the community, particularly through their experienced staff consisting of medical staff and various community leaders [57]. A bottom-up approach using local information providers is crucial to creating trust in the affected community and conducting a successful OCV campaign.

When there is delay of reporting of suspected and/or confirmed cholera cases from a lower to a higher surveillance level, there is a risk of outbreaks spiraling out of control. There may be a host of reasons for cholera cases not being reported. A country’s export interests and dependence on tourism have previously been perceived as possible reasons for not reporting cholera, with underreporting or no reporting as a consequence [58].

A finding of importance in several studies was the frequent absence of persons eligible for vaccination during OCV campaigns. To avoid this, OCV campaigns should be adapted to local contexts and involve flexible delivery strategies involving generous vaccination hours. Such planning was documented in a study on the implementation of an OCV campaign in South Sudan. Organisers adapted the vaccination delivery strategy to the local context, using a mixed approach of fixed sites and mobile door-to-door vaccination teams resulting in improved vaccination coverage [59].

The results of this article also show the crucial need of having laboratory capacity as close as possible to where cases occur, as a major facilitator for surveillance in many different settings and countries. A country may have a surveillance system in place, but without well-trained staff who are skilled in laboratory testing and diagnosis for confirmation of cholera cases, cholera surveillance cannot function properly. If there is an additional lack of sufficient laboratory resources and capabilities this will further hinder important surveillance activities. For instance, lacking diagnostic and laboratory capabilities hindered complete surveillance data on cholera in Nepal [60]. Specifically, Nepal’s shortage of experienced staff and persistent laboratory challenges capture the issue of lacking resources for proper cholera control [60].

Shortage of global OCV stockpiles also hinder interventions for cholera control, as OCV campaigns cannot be fully implemented when vaccine supply does not meet vaccine demand [61]. This issue is mirrored by the global discussion on vaccine availability in the case of the Coronavirus disease 19 (COVID-19) pandemic which brought issues on vaccine availability to the forefront of the global public health agenda. The paradox of having vaccines which can prevent a number of diseases, which in turn would aid large populations in avoiding disease and promote public health initiatives, while not being able to distribute said vaccines in an equitable manner will continue to be one of the crucial topics of public health in need of further research and pratical solutions.

Although the included journal articles on surveillance discussed the detection of cholera cases, there was little mentioning of hotspot mapping as an important part of surveillance activities. Hotspot mapping means identifying places where the likelihood of contracting cholera is elevated. Such mapping facilitates targeted OCV campaigns where there is a higher risk of an outbreak [5]. There is a higher chance of preventing and eliminating cholera by targeting cholera hotspots for control interventions, and having a strong surveillance system informs proper hotspot mapping [62]. However, it is our understanding that there is a challenge to limiting cholera surveillance to known hotspots or cholera endemic areas as the disease tends to migrate from one place to another. Therefore, while cholera hotspots should be prioritised for surveillance activities, say via a sentinel surveillance system, there is the need to broaden cholera surveillance in a country.

### Implications of findings for research, practice and policy

The results presented on the importance of clearly communicating OCV information to communities can inform future research on how to develop and adapt specific cholera and OCV information campaigns tailored to local contexts. There is a need for further studies on what information should be provided and how to get messages across to populations affected by cholera. The scoping review also supports the need for research on vaccine hesitancy, further supported by findings from the COVID-19 pandemic in reports of vaccine hesitancy from many developing countries [63]. Together with other studies on communicable diseases and their vaccines, there is an opportunity to share knowledge on how to prevent vaccine hesitancy and establish what mechanisms enable vaccine acceptance. Further research on cholera surveillance could also draw from facilitators and barriers found in this article, while examining how to improve timely reporting and access to resources for surveillance. For example, since mHealth was found to be a facilitator under resources and logistics, there should be more implementation of and research on digital health solutions for surveillance and innovative OCV delivery strategies. A study on mHealth solutions for the COVID-19 pandemic response in India and Vietnam focused on the use of applications to furher a public health response. These applications aided in contact tracing, telemedicine etc. The authors argued that although such applications can further communications and accessibility in LMIC settings, the point of accessibility to technology must always be considered in order to provide mHealth benefits in an equitable manner [64]. Another study on the use of mHealth in LMICs argued that although mobile phones are ubiquitous and there lies great promise in their use wihin healthcare, mHealth cannot function properly without well-established healthcare systems. Meaning that in order for mHealth solutions to be able to provide long-term value, there has to be a healthcare system reliable enough to connect with mHealth solutions [65].

Recommendations for the betterment of cholera surveillance efforts, based on the results of this article, include detailed and structured record-keeping and reporting, having proper tools and know-how to analyse surveillance data, potential use of GPS to pin-point accurate locations of cholera cases as well as showing flexibility and providing generous hours for vaccine campaigns in order to reach populations during more hours of the week. This could further chances of reaching people who are away for work during the day and are not available during regular vaccination hours. There is also room for improving communication between neighbouring communities in order to strenghten local-level collaboration. In order to increase communication there could be a focus on low-cost mHealth interventions, such as using designated cholera mobile phones in order to communicate new cases between neighbouring communities and sharing and providing assitance and information.

Cholera often appears in already vulnerable contexts, in places of conflict or crisis, increasing the burden of the disease. African and Asian countries are particularly affected by cholera and its consequences. Many of the issues relating to COVID-19 such as tracking cases and distributing vaccines while preventing vaccine hesitancy, mirror the issues faced by cholera control. Furthermore, cholera control activities are hampered due to limited focus and resources during the COVID-19 pandemic, increasing the risk of cholera transmission in vulnerable regions [66]. It is possible that successful OCV implementation can inform COVID-19 vaccine rollout by demonstrating how vaccines can be distributed to hard-to-reach populations [66]. Furhermore, as demonstraded by a study on COVID-19 vaccine equity and availability in LMICs, there is also a need for cholera control efforts to include the principles of equity along each stage of the vaccine development and distibution process [67].

Concerning the concept of vaccine information, COVID-19 and cholera prevention efforts may benefit from each other, or risk exacerbating vaccine hesitancy. It is crucial to inform people about vaccine function, importance, benefits, risks, safety and explain, in the case of OCV, that the vaccine is aimed at adults as well as children. The issue of vaccine hesitancy has become clear during the COVID-19 pandemic, with a study on vaccine acceptability in LMIC settings showing that 50% of LMIC residents were willing to accept the vaccine against COVID-19 [68]. The study also found, as did the results of this article, that engagement of communities and their leaders as well as training of healthcare professionals is vital in order to further knowledge of vaccines. There is also utility in using media as a way to campaign for increasing awareness of vaccines [68], which would be useful in different vaccine settings, whether it be cholera or COVID-19. There is also a need to involve community members in the development and dissemination of vaccine information. Governments and organisations have a responsibility to inform and take community members’ queries into account before any vaccination starts. Additionally, where there are lacking surveillance systems, hopefully a renewed focus on surveillance activities due to COVID-19, may influence cholera surveillance as well.

As demonstrated by the results of this article, collaboration between community members, cholera stakeholders and representatives from government bodies and NGOs is crucial for furthering surveillance and OCV campaign activities. Being a conglomerate of various stakeholders including cholera endemic countries and the WHO, the GTFCC is representing and striving for cooperation in the quest to eliminate cholera. Going forward, the GTFCC is bringing surveillance, OCV and cooperation to the forefront of their policymaking: strengthening proper surveillance as the key to early detection of outbreaks; using OCV to show that cholera is not an inevitable disease and presenting solutions to cholera hotspots and outbreaks; implementing Water, Sanitation and Hygiene (WASH) programmes; and providing support and resources for national stakeholders working to prevent cholera in their own local contexts [5]. Additionally, the findings of this scoping review could further inform surveillance and OCV policy change. On a global level, health organisations such as the WHO and the GTFCC, should focus more on providing actual resources for cholera interventions such as surveillance and OCV and develop more practical checklists based on established research findings. On a regional level, there should be more investment into communication networks for surveillance, facilitating proper cholera surveillance between different levels of reporting (local, regional, national and global). The regional level of policymaking should also include focusing on cholera education through information about the disease and existing vaccines. Furthermore, clear protocols for planning OCV campaigns should be developed and adapted to local contexts. Finally, on a national level, valuable change relating to the findings of this scoping review, would be investment in training of a growing number of well-trained staff for surveillance and OCV interventions. National policymakers should also actively include trusted community members and leaders in policy decisions on cholera control, as they have a direct connection the community.

### Strengths and limitations

Maintaining wide research questions facilitated the inclusion of a broad range of data and lessened the risk of overlooking relevant information. Furthermore, this scoping review covered a broad timeline, while capturing both historical and contemporary narratives of cholera surveillance and OCV. However, the review has some limitations that are worth noting. First, the use of three databases for the review of documents and restriction of searches to only documents written in English might have biased the findings if the omitted documents were systematically different from those included for the review. However, the duplications observed following Google search suggest this may not be a significant issue. Moreover, these databases were chosen in consultation with a librarian with extensive experience in conducting systematic reviews. The scoping review process does not necessarily put as much focus on the assessment of quality of included journal articles as a systematic review would [13]. By not assessing quality there is inherent risk of including documents of a lesser quality in the present review. Since choosing and analysing journal articles can be a subjective process, there is also a risk of a researcher bias. Following the eligibility criteria and using the research questions to guide the analysis were important steps taken to avoid researcher bias and there was a constant engagement between the lead researcher and co-authors during this iterative process. Another limitation worth noting is the limitation of detailed discussion on major issues, such as vaccine availability, within the scope of this article. This limitation further mirrors how there is often a lacking discussion on vaccine availability within published reports and documents on OCV. This is an issue that needs to be addressed in a robust manner in all research on OCV and other vaccines, as vaccine availability is key in order to be able to implement any OCV or vaccine interventions at all. We recommend further studies focusing on oral cholera vaccine availability in cholera endemic settings, especially context-specific barriers, using either a focus group discussion or key informant interviews with cholera stakeholders.

## Conclusion

Resources are crucial for timely and accurate cholera surveillance systems. OCV interventions could be more successful if knowledge of cholera and its vaccines are prioritised in communities, along with effective community engagement where people getting vaccinated can be reassured of vaccine safety by people they trust. Having proper resources and good planning and coordination, are important for linking surveillance to OCV interventions. Combining the GTFCC’s efforts with knowledge provided by active researchers will further move towards global elimination of cholera.

## Electronic supplementary material

Below is the link to the electronic supplementary material.

Supplementary Material 1

Supplementary Material 2

Supplementary Material 3

Supplementary Material 4

## Data Availability

The documents used for this study are available upon reasonable request to the corresponding author.

## Abbreviations

CINAHL: Cumulative Index to Nursing and Allied Health Literature
COVID-19: Coronavirus disease 2019
EPI: Expanded Programme on Immunization
GTFCC: Global Task Force on Cholera Control
IDP: Internally displaced persons
LMIC: Lower- and middle-income country
MeSH: Medical Subject Headings
NGO: Non-governmental organisation
OCV: Oral cholera vaccines
PCR: Polymerase chain reaction
PRISMA: ScR:Preferred Reporting Items for Systematic Reviews and Meta-Analyses – Extension for Scoping Reviews
RCT: Randomised control trial
SDGs: Sustainable Development Goals
WASH: Water, Sanitation, and Hygiene
WHO: World Health Organization
WTP: Willingness to Pay
