# Supplementary material for: A scoping review of facilitators and barriers influencing the implementation of surveillance and oral cholera vaccine interventions for cholera control in lower- and middle-income countries
Source: BMC Public Health. 2023 Mar 8;23:455. doi: 10.1186/s12889-023-15326-2 (PMC9994404; doi:10.1186/s12889-023-15326-2)
Supplement: Supplementary file 1 — Supplementary Material 1 [file 12889_2023_15326_MOESM1_ESM.docx]

**Additional file 1 Database search outputs**

| **Database** | **Output (including duplicates)** |
| --- | --- |
| PubMed | 5145 |
| CINAHL | 1025 |
| Web of Science | 1966 |
| **Total** | **8136** |
