# Supplementary material for: A scoping review of facilitators and barriers influencing the implementation of surveillance and oral cholera vaccine interventions for cholera control in lower- and middle-income countries
Source: BMC Public Health. 2023 Mar 8;23:455. doi: 10.1186/s12889-023-15326-2 (PMC9994404; doi:10.1186/s12889-023-15326-2)
Supplement: Supplementary file 2 — Supplementary Material 2 [file 12889_2023_15326_MOESM2_ESM.docx]

**Additional file 2 Database searches with search terms and outputs**

| **PubMed (1990 to date, English results) 12 February 2021** | **Number of results** |
| --- | --- |
| cholera vaccin*  [Title/Abstract] | 818 |
| cholera vaccines [MESH terms] OR cholera vaccin* [Title/Abstract] | 1181 |
| cholera [MESH terms] AND epidemiology [MESH terms] | 132 |
| cholera surveillance [Title/Abstract] | 60 |
| (cholera[MeSH Terms]) AND (surveillance[Title/Abstract]) | 333 |
| ((cholera surveillance[Title/Abstract]) OR (epidemiology[Title/Abstract])) AND (cholera[Title/Abstract]) | 415 |
| (cholera surveillance[Title/Abstract]) OR (epidemiology[Title/Abstract] AND cholera[Title/Abstract]) OR ((cholera[MeSH Terms]) AND (epidemiology[MeSH Terms])) | 510 |
| (cholera surveillance[Title/Abstract]) AND (cholera vaccines [MeSH Terms]) | 5 |
| cholera surveillance[Title/Abstract] AND cholera vaccines [Title/Abstract] | 2 |
| (cholera vaccines [MeSH Terms] OR cholera vaccin* [Title/Abstract]) AND ((cholera surveillance [Title/Abstract]) OR (epidemiology [Title/Abstract]) AND cholera [Title/Abstract]) | 39 |
| ((cholera vaccines[MeSH Terms]) OR (cholera vaccin*[Title/Abstract])) OR ((cholera surveillance[Title/Abstract] OR (epidemiology[Title/Abstract] AND cholera[Title/Abstract])) OR ((cholera[MeSH Terms]) AND (epidemiology[MeSH Terms]))) | 1650 |
| Total results from PubMed | 5145 (including duplicates) |
| **CINAHL (1990 to date, English results, using “suggest subject terms”, TI = Title, AB = Abstract) 12 February 2021** | **Number of results** |
| TI cholera vaccin* OR AB cholera vaccin* | 280 |
| TI cholera vaccin* | 151 |
| AB cholera vaccin* | 204 |
| AB cholera AND AB epidemiology | 52 |
| TI cholera AND TI epidemiology | 15 |
| TI cholera AND AB epidemiology | 25 |
| AB cholera AND TI epidemiology | 26 |
| cholera AND disease surveillance | 168 |
| AB cholera AND AB disease surveillance | 59 |
| TI cholera AND AB disease surveillance | 23 |
| AB cholera AND TI disease surveillance | 9 |
| AB cholera vaccine AND AB epidemiology | 5 |
| AB cholera vaccine AND AB disease surveillance | 8 |
| Total results from CINAHL | 1025 (including duplicates) |
| **Web of Science (1990 to date, English results, TI = Title, AB = Abstract) 12 February 2021** | **Number of results** |
| TI=(“cholera vaccin*”) OR AB=(“cholera vaccin*”) | 866 |
| TI=(cholera AND epidemiology) OR AB=(cholera AND epidemiology) | 288 |
| TI=(“cholera surveillance”) OR AB=(“cholera surveillance”) | 54 |
| TI=(cholera) AND TI=(surveillance) | 44 |
| AB=(cholera) AND AB=(surveillance) | 424 |
| TI=(cholera surveillance) AND TI=(cholera vaccin*) | 4 |
| AB=(cholera surveillance) AND AB=(cholera vaccin*) | 90 |
| AB=(“cholera surveillance”) AND AB=(“cholera vaccin*”) | 5 |
| AB=(cholera vaccin*) AND AB=(epidemiology) AND AB=(cholera) | 37 |
| TI=(cholera vaccin*) AND (TI=(cholera surveillance) OR TI=(cholera AND epidemiology)) | 4 |
| AB=(cholera vaccin*) AND (AB=(cholera surveillance) OR AB=(cholera AND epidemiology)) | 118 |
| TI=(cholera vaccin*) AND (AB=(cholera surveillance) OR AB=(cholera AND epidemiology)) | 32 |
| Total results from Web of Science | 1966 (including duplicates) |
| **Total results from all three databases** | **8136 (including duplicates)** |
