# Supplementary material for: A scoping review of facilitators and barriers influencing the implementation of surveillance and oral cholera vaccine interventions for cholera control in lower- and middle-income countries
Source: BMC Public Health. 2023 Mar 8;23:455. doi: 10.1186/s12889-023-15326-2 (PMC9994404; doi:10.1186/s12889-023-15326-2)
Supplement: Supplementary file 3 — Supplementary Material 3 [file 12889_2023_15326_MOESM3_ESM.docx]

**Additional file 3 Google searches with search terms and outputs**

| **Google 2 April 2021** | **Number of selected results for potential inclusion after screening first 10 pages according to eligibility criteria** |
| --- | --- |
| cholera + surveillance + epidemiology | 8 |
| **Google 5 April** | **Number of selected results for potential inclusion after screening first 10 pages according to eligibility criteria** |
| cholera + vaccines | 3 |
