# Supplementary material for: A scoping review of facilitators and barriers influencing the implementation of surveillance and oral cholera vaccine interventions for cholera control in lower- and middle-income countries
Source: BMC Public Health. 2023 Mar 8;23:455. doi: 10.1186/s12889-023-15326-2 (PMC9994404; doi:10.1186/s12889-023-15326-2)
Supplement: Supplementary file 4 — Supplementary Material 4 [file 12889_2023_15326_MOESM4_ESM.docx]

**Additional file 4 Included journal articles with summarised main results as well as subsequent themes from thematic analysis**

**Surveillance** themes – Timeliness and reporting (TR), resources and laboratory capabilities (RLC). **OCV** themes – Information and awareness (IA), Community acceptance and trusted community leaders (CAT), planning and coordination (PC), resources and logistics (RL). Interface between surveillance and OCV (ISO) in *italics*.

| **Study characteristics** | | | | | **Summarised main results** | | | |
| --- | --- | --- | --- | --- | --- | --- | --- | --- |
| **First author (published)** | **Setting and country** | **Study year** | **Study period** | **Study design** | **Surveillance facilitators** | **Surveillance barriers** | **OCV facilitators** | **OCV barriers** |
| Adjei EY (2017) | Accra, Ghana | Year 2014 | January 2014 using surveillance data from 2011-2013 | Evaluation | Well-trained staff (RLC). Case definition (RLC). |  |  |  |
| Amani A (2021) | North region, Cameroon | 2019 | August 1-5, 2019 (comparing with 2018) | Descriptive | *Proper surveillance (effect OCV) (ISO).* | *Lack of surveillance/reporting (hinder evaluation) (ISO).* | Community acceptance of OCV (CAT). Proper vaccine management (RL). | Stockpile shortage (RL). Timing vs two doses (core problem of vaccine shortage?) (RL). |
| Burnett EM (2019) | Arbonite, Centre & Ouest, Haiti | 2014 | 26 October 2014-11 November 2014 | Mixed methods |  |  | Knowing about vaccine activities (megaphones) (IA). | Not hearing about vaccine activities (IA). Absent during campaign (PC). |
| Bwire G (2013) | Uganda | 2007-2011 | 2007-2011 | Descriptive | Resources (financial/human) (RLC). Timeliness (system prepared 🡪early response) (TR). Collaboration (TR). | Weak laboratory capacity (district level) (RLC). Resource challenges (RLC). Missing information (national level?) (TR). Weak local-level collaboration (TR). |  |  |
| Bwire G (2020) | Hoima district, Uganda | 2018 | February 2018-June 2018 | Evaluation |  |  | Knowledge of OCV (prevents cholera) (IA). Existing OCV acceptance (CAT). Well-trained staff (RL). | Absent during campaign (PC). Unclear dates of 2nd round, unpredictable planning (shipping/customs) (PC). Vaccination team missing households (PC). |
| Démolis R (2018) | Nampula, Mozam-bique | 2016 | June 2016 | Mixed methods |  |  | Seeing disease as serious (wanting vaccine) (IA). Clear communication of key campaign messages (IA). Positive attitude (towards vaccines in general) (CAT). | Lack of knowledge (benefits/importance of OCV) 🡪 hesitancy (IA). Hesitancy 🡪 oral vs injection (oral may be perceived as having less efficacy) (IA). Community-level rhetoric: use of OCV campaign of enemy/political opponent (CAT). |
| Fatiregun AA  (2013) | Sabo community of Ife, Osun state, Nigeria | 2010 | August 2010-September 2010 | Evaluation |  | Resources (technology) (RLC). Poor record-keeping (TR). Incomplete reporting (TR). Poor knowledge of reporting system (TR). |  |  |
| Grandesso F (2018) | Lake Chilwa region, Malawi | 2016 | March 2016-April 2016 | Evaluation |  |  | Campaign to remind of self-administering 2nd dose (IA). Thermal stability (self-administering out of cold chain) (RL). Practical/convenient delivery of 2nd dose of vaccine (when self-administering) (RL). Self-administering found practical/convenient (RL). | Innovative adminstering strategy not understood (poor communication) (IA). Absent during campaign (PC). Self-adminstering seen as complicated (RL). Vaccine not available at vaccination post (RL). |
| Heyerdahl LW (2018) | Lake Chilwa region, Malawi | 2016 | January 2016-March 2016 | Evaluation |  |  | Existing knowledge on OCV importance (protects) (IA). Correct information on self-adminstering (IA). Existing willingness to get OCV (CAT). Well-trained community leaders (trust?) (CAT). Designing administering of OCV way the public responds to (PC). | Incorrect knowledge on OCV 🡪 refusal (IA). Uncertain/not trust vaccine (IA). 2nd dose strategies not communicated (dates/sites) (IA). Self-administering: worrying about storage (at home) (RL). |
| Heyerdahl LW (2019) | Lusaka, Zambia | 2016 | April 2016-December 2016 | Evaluation |  |  | Use of local community channels (list) (believed their sources) (CAT). Use of social networkds 🡪 provide information on OCV (CAT). OCV campaign on weekend/mobile 🡪 reach (PC). | Not clearly communicating information on cholera (IA). Lacking vaccine information on side effects/who should take it/level of duration of protection (if persons would then see side effects, credibility would be given to other persons spreading rumours of OCV’s lack of safety (IA). Bad taste (CAT). |
| Kar SK (2014) | Satyabadi block, Puri district, Odisha, India | 2011 | May 2011-June 2011 | Inter-vention study |  |  | Micro-planning (PC). Planning delivery in feasible way (PC). Human resources (RL). Having available cold chain (RL). Pre-existing functioning health system (logistic/polio) (RL). | Perception of vaccine as only for children (IA). Complicated packaging of vaccine vial (RL). Cold chain requirements (difficult in hot and humid conditions) (RL). |
| Kar SK (2014) | Odisha, India | 2011 | August 2011-September 2011 | Evaluation |  |  | Knowledge of cholera symptoms (IA). Community: informed stakeholders (CAT). | Insufficient information on OCV (IA). Bad taste/smell (CAT). |
| Khan AI (2019) | Dhaka, Bangladesh | 2017 | March 2017-April 2017 | Inter-vention study |  |  | Existing positive attitudes towards vaccines in general (positive impact on other vaccine campaigns (CAT). Cooperation between actors (PC). 2nd dose self-administering seen as practical/convenient (RL). Self-administering lowers logistical burden (RL). Keep OCV correctly at home (refrigerated) (RL). | Bad taste (CAT). Hard to reach working adults (PC). Difficulty taking vaccine themselves (RL). |
| Lam E (2017) | IDP (Internally Displaced Persons) camps, Iraq | 2015 | October 2015-December 2015 | Evaluation |  |  | Information about campaign (IA). Combining ways of campaign (mobile/door-to-door, adapting) (PC). Adaptive vaccine strategies (PC). Rapidity of OCV response activity (detection 🡪 implementation of campaign) (PC). Use of stockpole (RL). Committed staff/partners (RL). | Lack of faith in OCV (IA). Absent during campaign (PC). Vaccination teams not visiting (PC). Unavailability of vaccine (RL). |
| Lubogo M (2020) | 9 target districts, Somalia | 2017 | March 2017-October 2017 | Evaluation |  |  | Information about vaccine campaign (IA). Using accepted community volunteers/leaders (despite insecurity) (CAT). Social mobilization (experienced polio mobilizers, informing of need of two doses) (CAT). Use of existing EPI/PEI structures (RL). | Absent during campaign (PC). Vaccination team not visiting (PC). |
| Luquero FJ (2013) | Boffa & Forécariah, Guinea | 2012 | April 2012-June 2012 | Evaluation |  |  | Knowledge on cholera (motivator) (IA). High awareness of campaign (IA). Awareness campaign (reach) (IA). Would be vaccinated in future (CAT). High willingness to get vaccinated again (CAT). Reassuring that Ministry of Health etc. participated (CAT). Vaccinating from early to late (reach) (PC). | Bad taste (leading to incomplete doses, Dukoral) (CAT). Absent during campaign (PC). Not having time/being busy at time of 2nd dose (PC). |
| Massing LA (2018) | Kalemie, Democratic Republic of Congo | 2014 | August 2014 | Evaluation |  |  | Being aware of campaign (IA). Would be vaccinated in future (CAT). Bad taste (CAT). | Felt vaccine had no effect, cause illness (IA). Refusal due to religion/traditions, seen as unsafe (IA). Vaccine seen as for children (IA). Absent during campaign (many men, at work) (PC). |
| Merten S (2013) | Katanga province, Democratic Republic of Congo | 2010 | August 2010-September 2010 | Mixed methods |  |  | High acceptance of OCV (past experience, awareness/fear) (IA). Vaccination considered most efficient prevention of cholera (IA). |  |
| Msyamboza KP (2016) | Nsanje district, Malawi | 2015 | March 2015-April 2015 | Evaluation |  |  | OCV accepted by community (CAT). Rapid conducting of first round after decision (PC). Use of existing cold chain (EPI for storing vaccines) (RL). | Absent during campaign (PC). |
| Ngwa MC (2020) | Borno state, Nigeria | 2018 | February 2018 | Mixed methods |  | *Estimating who needs vaccines (hindered by lacking records/population estimates) (ISO).* | Awareness of OCV as protection against cholera (IA). Acceptability of OCV (high-risk populations in camps/conflicts) (CAT). Main information source (list, bottom-up) (CAT). | Absent due to travel/work (door-to-door campaign reaching more women/people at home, less men at work) (PC). |
| Ngwa MC (2020) | Borno state, Nigeria | 2018 | February 2018 | Evaluation | Phone/technology (detect and notify) (RLC). Coordination (prioritization) (TR). | Lack well-trained staff (RLC). Lack laboratory capability (RLC). Delayed declaration (because of other) (TR). | High advocacy for OCV safety from commissioner (CAT). Using/adapting microplanning context to context (from Sierra Leone to Nigeria) (PC). Approving OCV for endemic, then being prepared for outbreak (PC). Well-coordinated (PC). Fast planning/coordination 🡪 deploy OCV (PC). Experienced staff (RL). Use of polio mechanism (experienced staff, logistic support) (RL). Batches/transdocking (getting over logistical hurdle) (RL). | Challenge to use polio platform (vaccine seen as only for children, reluctance) (IA). Challenge to use polio platform (vaccination cards not distributed during 1st round because of assumption there would only be one round) (RL). Challenge to use polio platform (polio vaccinators not familiar with opening vials) (RL). Poor communication networks (hinder data flow, hard to inform of needed actions) (RL). Underbudget of 1st round (extra work, less pay, was sorted by 2nd round) (RL). Lack of bank accounts on ward level (financing vaccinations, vaccinators had to carry money) (RL). Polio cold chain limitations/timeliness challenges (RL). |
| Ngwa MC (2016) | Far North & Centre regions, Cameroon | 2013 | April 2013-June 2013 | Descriptive | Well-trained staff (RLC). Good laboratory capability (reference laboratory when no district laboratory) (RLC). Resources (technology/vehicles – these help with surveillance functions) (RLC). Resources (technology) (RLC). Phone/technology (to motivate reporting) (RLC). Phone/technology (coordinate) (RLC). Phone/technology (report) (RLC). | Lack staff/technology (RLC). Lacking laboratory capability (RLC). Local – lack knowledge of surveillance (TR). Absence (identify male cases) (TR). |  | No vaccine stocks (RL). |
| Noora CL (2017) | Brong Ahafo region, Ghana | 2014 | July 2014-December 2014 | Descriptive |  | Laboratory problem (samples) (RLC). Laboratory samples (only regional) (RLC). Delay from lower to higher level (not effective) (TR). Inaccurate location (lack district hospital, cases) (TR). |  |  |
| Ohene S (2016) | Akatsi district (Volta region) & Komenda-Edina-Eguafo-Abirem (KEEA) municipal (Central region), Ghana | 2012 | September 2012-November 2012 | Descriptive | Timeliness (early reporting, confirm and respond) (TR). | Lacking community-based surveillance (late) (TR). Discrepancy/inaccuracy in reported cases (national level) (TR). Incomplete data analysis (TR). Lack neighbour communication (TR). |  |  |
| Peprah D (2016) | Juba, South Sudan | 2014 | February 2014-April 2014 (when campaign took place, study took place four months later) | Interview study |  |  | High fear of disease (having knowledge, seen as serious) (IA). OCV campaign positively perceived (confidence it prevented) (IA). Hope to be vaccinated again (CAT). Recommend others to get vaccinated (CAT). Most would recommend others (CAT). Increased trust in vaccine providers (CAT). | Distrust of authenticity of vaccine, seeing adverse events following immunization (IA). Questioning dose recommendations despite awareness of need of two doses (inaccurate ideas, enough with one dose, want more doses) (IA). Bad taste/smell (CAT). Busy/absent during campaign (PC). |
| Phares CR (2016) | Maela, Thailand | 2005-2013 | 2005-2012; January 2013-March 2013 | Evaluation | *Timing campaign to off season (thanks to surveillance) (ISO).* |  | Trusted organisation (familiar source of information/healthcare) (CAT). Shanchol affordable/easy (RL). | Bad taste (CAT). Lower 2nd dose because of competing activities (PC). Cold chain requirements (supplies needed, complicated campaign logistics) (RL). |
| Poncin M (2018) | Lusaka, Zambia | 2016 | April 2016 | Evaluation | Timeliness (detect and declare) (TR). | *Dynamic population, hard to estimate, limits accuracy of administrative coverage (ISO).* | Fast response/coordination (mass OCV quickly after OCV decision) (PC). Good local epidemiology + collaboration 🡪 vaccine choice and request 🡪 quick start of campaign (PC). Thermostability 🡪 cold chain/logistics/vaccine distribution made easier (RL). | Insufficient available doses (2nd dose hinder) (RL). |
| Porta MI (2014) | Maban county, South Sudan | 2012-2013 | December 2012-February 2013 | Evaluation |  |  | High OCV acceptance (eager) (CAT). Social mobilization (essential informing population about vaccine, use community health workers who knew camp (CAT). Fixed sites (more people vaccinated in shorter time, fewer human resources) (PC). Door/mobile (easier reaching whole population but requires more human resources (PC). Mobile/door (well-adapted to context) (PC). Phased storage/release (helped when single-dose vial needed high storage volume) (RL). Thermostability of Shanchol (less cold chain) (RL). | Vaccination card challenges (RL). OCV not user-friendly (RL). Single-dose needs large storage in cold chain (hard in humanitarian setting) (RL). Cold chain ruptures (freezing, loss of vials) (RL). |
| Schaetti C (2012) | Peri-urban Shehia of Chumbuni in Unguja & rural Shehia of Mwambe in Pemba, Zanzibar (Tanzania) | 2009 | June 2009-July 2009 | Mixed methods |  |  |  | Fear of side effects, doubt of OCV effectiveness (IA). Competing obligations/priorities + less likely to get timely information on vaccine campaign 🡪 no vaccination (IA). |
| Semá Baltazar C  (2018) | Nampula, Mozam-bique | 2016 | October 2016 | Mixed methods |  | *Lack map (hindering exhaustive door-to-door campaign) (ISO).* | Knowledge of cholera (IA). Reassurance of safe vaccine (community leader) (CAT). Vaccination cards for 2nd dose (RL). Good (logistical) management of vaccines (cold chain) (RL). | Insufficient information about campaign (date/time) (IA). Bad taste (CAT). Insufficient planning (PC). |
| Semá Baltazar C (2017) | Beira city, Mocuba district & Pemba city, Mozam-bique | 2011-2015 | October 2011-December 2015 | Mixed methods | PCR test capability (RLC). | Lack laboratory capability (RLC). Limited resources (RLC). Limiting surveillance (some zones) (TR). |  |  |
| Sharp A (2020) | South department, Haiti | 2017 | June 2017-July 2017 | Evaluation |  |  | Hearing of campaign (local criers, shows importance of community engagement and mobilization (CAT). Useful mixed approach to vaccination campaign (reach) (PC). | Absent during campaign (PC). |
| Teng JE (2014) | Bocozel & Grand Saline, Haiti | 2012 | April 2012-June 2012 | Inter-vention study | *Nightly review 🡪 plan 🡪 adapt (ISO). Daily monitoring (case-finding) higher follow-up (ISO).* |  | mHealth (tablet/barcodes) - less print/manual (RL). Vaccine registry on each tablet (present at any vaccination post) (RL). |  |
| Tohme RA (2015) | Petite Anse & Cerca Carvajal, Haiti | 2013 | September 2013 | Evaluation |  |  | High awareness of campaign (IA). Information source: social mobilizer (megaphones), healthcare workers, family/friends (CAT). | Bad taste (spit out) (CAT). Absent during campaign (PC). |
| Uddin J (2014) | Dhaka, Bangladesh | 2011-2012 | May 2011-June 2012 | Descriptive |  |  | High interest to get OCV (believed OCV prevent cholera), great enthusiasm (IA). High acceptance of OCV (Bangladeshi people had positive impression of vaccines (had prevented infectious diseases through EPI)) (CAT). Recommend others to get vaccinated (CAT). Seen as safe because government implemented the vaccination (CAT). | Bad taste/smell (CAT). Some reluctant 🡪 due to a rumour that Bangladeshi people used as guinea pigs to test OCV – but rumour did not have much effect since policy-level people of the Ministry of Health (had?) strong support (CAT). Absent during campaign (PC). |
| Wahed T (2013) | Dhaka, Bangladesh | 2010-2011 | December 2010-February 2011 | Mixed methods |  |  | Agreed cholera could be prevented through vaccination (IA). Knowing OCV is available from NGOs) (IA). Highly positive attitude to OCV (CAT). | Few had heard of OCV (IA). |
